# Disability and health outcomes – from a cohort of people on long-term anti-retroviral therapy

**DOI:** 10.1080/17290376.2018.1459813

**Published:** 2018-04-10

**Authors:** Hellen Myezwa, Jill Hanass-Hancock, Adedayo Tunde Ajidahun, Bradley Carpenter

**Affiliations:** a Department of Physiotherapy, Faculty of Health Sciences, University of the Witwatersrand, 7 York Road, Parktown, Johannesburg, South Africa; b School of Health Science, Westville Campus, University of KwaZulu-Natal, Durban, South Africa; c South African Medical Reesarch Council, 123 Jan Hofmeyer Road, Durban, South Africa

**Keywords:** HIV, disability, depression, South Africa, adherence, antiretroviral

## Abstract

Human-immunodeficiency virus (HIV)/Acquired immunodeficiency Syndrome (AIDS) remains a major health problem in South Africa – even after two decades since the introduction of antiretroviral therapy (ART). Long-term survival with HIV is associated with new health-related issues and a risk of functional limitation/disability. The aim of this study was to assess functional limitation associated with HIV/AIDS among people living with HIV (PLHIV) in South Africa. This study is a cross-sectional survey using a cohort in an urban area in Gauteng province, South Africa. Data were collected using questionnaires through an interview process. The information collected included aspects such as demographics, livelihood, the state of mental and physical health, adherence and disability. A total of 1044 participants with an average age of 42 ± 12 years were included in the study, with 51.9% of the participants reporting functional limitations (WHODAS ≥ 2). These were reported mainly in the domains of participation (40.2%) and mobility (38.7%). In addition, adherence to ART, symptoms of poor physical health and depression were strongly associated with their functional limitations/disability. HIV as a chronic disease is associated with functional limitations that are not adequately addressed and pose a risk of long-term disability and negative adherence outcomes. Therefore, wellness for PLHIV/AIDS needs to include interventions that can prevent and manage disability.

## Introduction

Human Immunodeficiency Virus (HIV) and Acquired Immunodeficiency Syndrome (AIDS) remain key priority health areas in HIV-endemic countries in spite of the 30-year trajectory of this disease (Valdiserri, 2011). South Africa has one of the highest numbers of infected adults with HIV and in 2015, it was estimated that 7,000,000 people were living with HIV in South Africa (UNAIDS, 2014b). Owing to life-saving antiretroviral treatment (ART), more people are currently surviving than was the case previously. As a result, the focus of HIV management has shifted in recent years from that of a deadly disease towards that of managing a chronic condition (Deeks, Lewin, & Havlir, 2013). Managing chronicity requires a move towards a more integrated approach of treatment, care and support that includes rehabilitation in the continuum of care (Deeks et al., 2013; Myezwa, Stewart, Musenge, & Nesara, 2009; Nixon, Hanass-Hancock, Whiteside, & Barnett, 2011). This approach shows similarities with the systems thinking approach proposed by Valdiserri in his call for improved focus on demographic and epidemiological data in order to develop high-quality systems of care (Valdiserri, 2011).

There is very little understanding of the long-term health outcomes of living with chronic HIV in Africa and the related services needed to manage the associated health needs of patients after long periods on ART (Deeks et al., 2013; Hanass-Hancock, Myezwa, & Carpenter, 2015; Nixon et al., 2011). Co-morbidities such as tuberculosis (TB), cancer and depression have already received some attention (Gonzalez, Batchelder, Psaros, & Safren, 2011; Pawlowski, Jansson, Sköld, Rottenberg, & Källenius, 2012; Robbins, Shiels, Pfeiffer, & Engels, 2014). However, researchers on HIV and its chronicity have even less understanding on its long-term effects beyond the results emanating from the investigation of HIV-related conditions and co-morbidities.

Recent evidence has suggested that people living with HIV (PLHIV), including those on ART, experience a diverse set of disabilities (Banks, Zuurmond, Ferrand, & Kuper, 2015; Hanass-Hancock, Regondi, Van Egeraat, & Nixon, 2013; Health Canada, 2009; Myezwa et al., 2009; Myezwa, Buchalla, Jelsma, & Stewart, 2011). The United Nations (UN) Convention on the Rights of Persons with Disabilities (CRPD) has been instrumental in many policy debates, including those on HIV/AIDS. The CRPD offers a tool to protect and promote the rights of people with disability (PWD) but also of those in need of rehabilitation because they are affected by chronicity.

The intersection of disability with HIV requires a careful exploration of the impact of the virus and treatment from a multi-systemic perspective. There is, however, a dearth of empirical information on the intersection of HIV with disability to inform policy and practice. Understanding the scope, types of disabling effects, as well as HIV’s impact on health outcomes and treatment adherence, is crucial to inform the management of HIV as a chronic condition (Deeks et al., 2013; Hanass-Hancock et al., 2015). Existing literature used the International Classification of Function (ICF)^1^ as a lens to understand the relationship between HIV and disability (Banks et al., 2015; Hanass-Hancock & Nixon, 2009; Hanass-Hancock et al., 2013; 2015; Myezwa et al., 2009, 2011; Van As, Myezwa, Stewart, Maleka, & Musenge, 2009).

Studies using the ICF lens indicate a high burden of disability with diverse conditions among PLHIV and those on ART. However, we have little quantitative evidence from representative cohort studies that can better predict the scope and types of disability in populations living with HIV, and its impact on health and ART adherence.

The HIV-Live Study, collaboration between the Health, Economics, HIV and AIDS Research Division (HEARD) and the University of the Witwatersrand, Johannesburg, South Africa, sought to address this gap by investigating the disabling experiences in three different cohorts and settings. The study investigated the intersection of functional limitation as a proxy for disability, other health outcomes, treatment adherence and livelihood outcomes.

A recent publication reported the results of HIV disability and associations with health outcomes in the first cohort study (HIV-Live) in a semi-rural area in KwaZulu-Natal (KZN) which is considered the epicentre of the pandemic in South Africa (Hanass-Hancock et al., 2015). The results of the second cohort, in the urban setting of Gauteng (second-highest prevalence in the country), are presented in this paper. The main aim of the study was to investigate the scope and types of functional limitations/disability and their association with HIV-related health symptoms, including mental health and adherence to ARTs. The specific objectives were to describe the socio-demographical attributes, livelihood and medical history of the patients, as well as their physical, functional and mental health in relation to the presence of disability.

## Methods

A framework combining disability and livelihood formed the theoretical basis to investigate the relationship between disability and other outcomes in a cohort of people on ART in South Africa. The overall methodology was developed to include a cross-sectional survey in three cohorts: the first living in a semi urban area in KwaZulu-Natal (longitudinal observational study) and the other two in the urban area of Gauteng (South Africa) and the United States of America. The cohort from Gauteng included HIV patients being treated at the Helen Joseph Hospital Themba Lethu HIV clinic,^2^ the largest ART site in South Africa.

Each participant was approached consecutively during their routine visits for treatment review and the collection of their medication. All patients were routinely screened for height, weight, body mass index (BMI), blood pressure and screened further by a doctor. Participants had to be attending the outpatient HIV clinic, be between the ages of 18–65 years and have been on ART for six months or longer. Exclusion criteria included participants with any acute opportunistic infections such as active TB or pneumonia, and any patient who was pregnant and with severe mental impairment, as this would have impacted the disability measure.

A trained research assistant, who herself was a patient attending this clinic, approached the patients during their visit, explained the purpose of the study and sought initial permission to interview the patient. Three trained research assistants, including the principal investigator, collected data. All three were trained clinicians and could explain the concepts should misunderstandings arise. Two private rooms were available for conducting the interviews and arrangements were made for the patients to keep their place in the queue. The questionnaires were translated into isiZulu (a widely spoken language in South Africa) so that the researchers could use the standardised translated questionnaire where needed. The patient file was accessed after permission was granted by the patient. The length of time on ART and the presence of recent opportunistic infections were extracted, as well as the most recent CD4 count. The information extracted was verified with the patient in the interview. Ethics approval was obtained from the Human and Research Ethics Committee at the University of the Witwatersrand, Johannesburg, South Africa, and the written informed consent of the participant was obtained.

The estimated sample size for this study was calculated at 1050 with Stata 12.1 with a one-sample comparison of proportions (a one-sample-sized computation), a 90% power and two-sided test and an alpha level of 5%, with a hypothesised 55% of the population with disability (Myezwa et al., 2009, 2011). This is how the estimated sample size of 1050 was arrived at.

The data collection tool was developed using literature sources (Hanass-Hancock & Nixon, 2009; Hanass-Hancock et al., 2015; Myezwa et al., 2009, 2011; Nixon et al., 2011; Petersen, Hancock, Bhana, & Govender, 2013, 2014; Van As et al., 2009) as well as established and validated scales (see Table 1). Included in the survey tool, were demographics, biomarkers and scales for livelihood capital (human, financial, social, natural and physical capital), the patients’ health and medical history, functional limitations/disability, symptoms of depression and adherence to treatment.

**Table 1.** Outline of the psychometric properties of instruments used in this study.

Element measured	Tool	Variables	Literature source, reliability and validity	Data analysis
Socio-demographic	Socio-demographic questionnaire	Age gender and marital status (* other factors are indicated in the livelihoods section)	–	Descriptive and correlations
Livelihood	HIV-live livelihood capitals (self-developed from literature)	Education and financial capital, including source of income, physical and natural capital including housing, source of income, water and sanitation, social capital and food security	Livelihood – .728 Physical & natural capital – .699 Social capital – .908 Food security – .790 (Hanass-Hancock, Misselhorn, Carpenter, & Myezwa, 2016)	Descriptive
Medical history	Medical symptoms questionnaire	Confusion, Memory loss, Breathlessness, Fatigue, Diarrhoea, Nausea, Stomach Pain, Headache, Change in taste, Skin itching/changes. Muscular pain, Heartburn, Sore mouth, Vomiting, Fever, Kidney stones. Included in this section were the details of the weight and height of the patient, CD4 count	Duran et al. (2001)	The presence and the absence of each symptom were recorded as ‘Yes’ and ‘No’ respectively. The ‘Yes and ‘No’ responses was coded as ‘1’ and ‘0’, respectively. The sum of the symptoms was converted into percentages. Overall score converted to a metric of 0- 100% where 100 = full health and 0 experiencing all symptoms.
Physical and functional health	WHODAS World Health Organisation: disability assessment scale 2.0 (5 point Likert scale)	Mobility (standing and walking) Self-care (washing, getting dressed) Participation (involvement in life situations) Cognition (learning concentration and memory) Getting along (dealing with people and maintaining friendship) Life activities (work and education)	Internal consistency reliability was 0.94 (Cronbach’s alpha). It showed moderate convergent validity with EQ5D and RAND-12 (0.41–0.76) (Carlozzi et al., 2015)	WHO item response theory-scoring and data analysis system was used and the analysis uses overall weighted score of 36, 36 = lowest level of function. Cut off for presence of disability was set at ≥2 (Hanass-Hancock et al., 2015).
Mental health	CESD-10 (4 point Likert scale)	Ten questions prompting status in terms of: depression, level of bother, how fearful, hopeful, happy or lonely one is and the effort required to get going	The CES D-10 showed good internal consistency reliability with the original CES D 20 (*α* = 0.88 (Myer et al., 2008; Zhang et al., 2012)	A sum of the scores was taken and a cut off of 16 and above was considered at risk of depression. A converted summary score is calculated with a metric of 0–100 (100 is equal to no symptoms of depression and 0 depicts severe symptoms of depression).
Adherence	CASE adherence index	Three unique adherence questions combined to form a composite score	CASE adherence index showed strong correlation with the three day self-reported adherence (ROC curve >0.86, *p* < .001) (Mannheimer et al., 2006).	A sum of the scores was calculated out of 16, with scores below 11 indicating adherence issues.

**Fig. 1.**
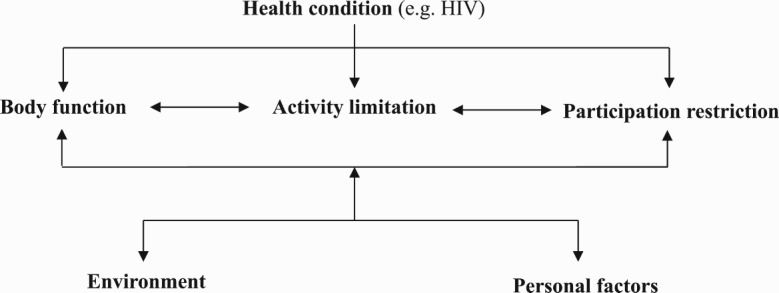
ICF conceptual framework.

The ICF was used as a conceptual framework to define disability (Fig. 1). The ICF defines disability as an umbrella term for impairments, activity limitations and participation restrictions. In addition, it includes the interaction between persons’ health condition and their context, the latter including their environmental and personal factors as part of the concept of disability.

The central element of disability is, therefore, the experience of functional or activity limitations on the basis of the interaction between changes in body function (impairment) and unaccommodating environments. As such, scales to investigate function and activity limitations were included in the tool and assessed through the World Health Organisation, a Disability Assessment Schedule (WHODAS) 2.0 scale (Üstün, Chatterji, Bickenbach, Kostanjsek, & Schneider, 2003). Importantly, their health condition was also measured using health symptoms commonly experienced by PLHIV/AIDS as explained in Table 1 (Duran et al., 2001). Adherence was measured using Mannheimer’s CASE adherence index (Mannheimer et al., 2008).

Table 1 outlines the instruments used in this study, the variables measured, along with the psychometric properties of the instruments and the data analysis approach. The Shapiro Wilk test was conducted and the data were non-linear for the main outcome measures (*p* < .001).

Descriptive statistics of frequency, percentage and median (interquartile range) were used to summarise the socio-demographic and clinical outcomes of the participants. Subsequently, Kendall’s Tau and Mann–Whitney *U* tests were used to examine the relationship between the independent and dependent variables (functional impairment, adherence and mental health). While Kendall’s Tau is not directly comparable to Pearson’s *r*, as it provides a smaller coefficient, it can be converted using a formula from Walker (2003). Multivariate linear regression was used to determine the predictors of the disability and secondary outcomes of adherence and mental health. Further analysis was conducted using multivariate linear regression to determine the relationship between primary outcome (functional limitation) and secondary outcome measures (mental health and adherence). All data analysis was done using the IBM SPSS 22.0.

## Results

### Socio-demographic outcomes

A total of 1044 participants were enrolled in the study: 71.8% were females and 28.1% were male with an average age of 42 ± 12 years. The average (median) WHODAS weighted score in this population was 0.5 ± 8, and 51.9% (542) of the participants reported disability (WHODAS ≥ 2). The average income was 5260 South African Rand across three months and 84.6% of the participants were found to have at least some form of high school education. Only a few participants reported having no source of income (*n* = 43) and no formal schooling (*n* = 17). The socio-demographic characteristics are outlined in Table 2.

**Table 2.** Socio-demographic characteristics and clinical outcomes of the participants.

Characteristics	Total (*n* = 1044)	People WHODAS 0–1 (*n* = 502)	People WHODAS ≥ 2 (*n* = 542)	*p*
Age, median (IQR) years	42(12)	42(11)	42(12.3)	.53
Age category, *n* (%)				
18–29	68(6.5)	33(6.6)	35(6.5)	
30–39	335(32.1)	164(32.7)	171(31.5)	
40–49	459(44)	223(44.4)	236(43.5)	
50–59	168(16.1)	76(15.1)	92(17)	
60+	14(1.3)	6(1.2)	8(1.5)	
*Gender, *n* (%)				<.001
Men	291(28.2)	164(32.7)	127(23.5)	
Women	750(71.8)	337(67.3)	413(76.5)	
Marital Status, *n* (%)				.06
Never married, single,	513(49.1)	235(46.8)	278(51.3)	
currently married,	250(23.9)	125(24.9)	125(23.1)	
divorced or widowed,	208(19.9)	97(19.3)	111(20.5)	
cohabiting	73(7)	45(9)	28(5.2)	
*Educational status, *n* (%)				.14
No formal schooling	17(1.6)	10(2)	7(1.3)	
Some primary school	143(13.7)	80(16)	63(11.6)	
High school	769(73.7)	367(73.3)	402(74.2)	
Post high school	113(10.8)	44(8.8)	69(12.7)	
Refused/Don’t know	1(0.1)	–	1(0.2)	
*Source of Income, *n* (%)				.79
Earned Income	604(58.3)	286(57.5)	318(59)	
Disability Grant	14(1.4)	–	14(2.6)	
Other Grants	77(7.4)	19(3.8)	58(10.8)	
No Income	43(4.2)	30(6)	13(2.4)	
Refused	197(19)	122(24.5)	75(13.9)	
Other income	94(9.1)	38(7.6)	56(10.4)	
Gifts	7(0.7)	2(0.4)	5(0.9)	
Income, median (IQR)	5260.6(9000)	4500(9000)	3600(6540)	
WHODAS weighted score, mean out of 36 (±SD)	0.5(8)	0(0)	8(9)	<.001
CES-10 Score, mean out of 30 (±SD)	11(9)	10(10)	12(10)	<.001
*Adherence score, median (IQR)	16(2.8)	16(1)	14.2(3)	<.001
Adherence score above 10, *n* (%)	148(14.2)	65(13)	83(15.4)	
Adherence score of 10 or less, *n* (%)	893(85.8)	436(87)	457(84.6)	
Health Symptoms, mean out of 16 (±SD)	5(6)	4(5)	6(5)	<.001
*BMI, median (IQR)	25(8.1)	24.9(7.5)	25.2(8.6)	.37
Normal	432(42.7)	245(44.4)	187(40.7)	
Underweight	73(7.2)	36(6.5)	37(8)	
Overweight	273(27)	155(28.1)	118(25.7)	
Obese	234(23.1)	116(21)	118(25.7)	
*CD4 Count, *n* (%)				.25
<200	762(77.5)	366(77.9)	396(77.2)	
≥200	221(22.5)	104(22.1)	117(22.8)	
*Years of living with HIV since diagnosis, median (IQR)	8(6)	7(6)	9(6)	<.001
*Years on ART, median (IQR)	6(5)	6(5)	7(6)	.01

**Fig. 2.**
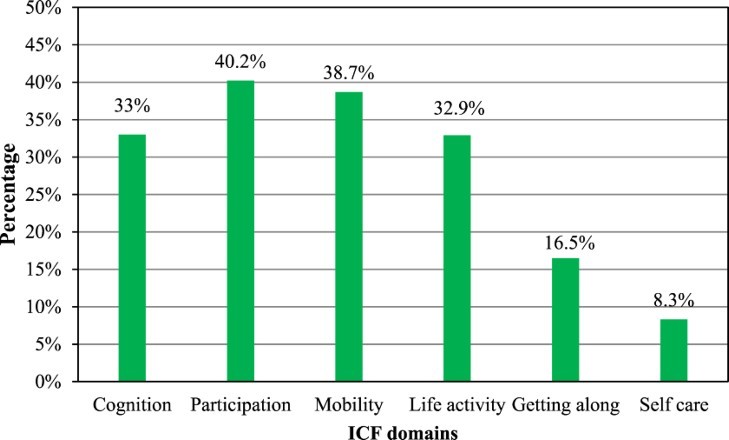
Functional limitations in the six domains.

Functional limitations as a proxy for disability were reported in all of the six domains (cognition, participation, mobility, life activity, getting along and self-care), with a higher prevalence in the participation (40.2%), mobility (38.7%), cognition (33%) and the life-activity domains (32.9%) as shown in Fig. 2.

### Relationship between functional limitations, health outcomes and socio-demographics

Gender $\left(r_{\tau b}=0.10,\;\;p<.001\right)$ and exposure to shock $\left(r_{\tau b}=0.32,\;\;p\;<.001\right)$ respectively showed significant associations with functional limitations. Similarly, the duration of HIV and the duration on ART from the time of diagnosis respectively showed significant associations with functional limitations (*p* < .001). All other socio-demographic variables showed no significant association with functional limitations, p>.05. The correlation analysis (Kendall Tau b) showed a positive weak correlation between functional limitations and outcome measures of physical health symptoms $\left(r_{\tau b}=0.21,\;\;p<.01\right)$ and mental health scores $\left(r_{\tau b}=0.19,\;\;p<.01\right)$ respectively, whereas, there was a negative weak correlation between the adherence score and the WHODAS scores $\left(r_{\tau b}=-0.15,\;\;p<.01\right)$. Table 3 shows the result of the correlation analysis between the independent and dependent variables.

**Table 3.** Relationship between socio-demographics and health outcome measures.

	WHODAS		Adherence		Mental health	
Variable	$r_{\tau b}$	*p*	$r_{\tau b}$	*p*	$r_{\tau b}$	*p*
Age	0.04	.12	0.02	.36	0.10	<.001
Gender	0.10	<.001	0.04	.17	0.09	<.001
Marital status	−0.04	.15	−0.03	.19	0.01	.73
Education	0.05	.05	−0.12	<.001	−0.13	<.001
Income	0.03	.19	−0.00	.90	−0.22	<.001
Years on ARVs	0.07	.01	−0.05	.07	0.04	.06
Years of living with HIV from time of diagnosis	0.07	<.001	−0.05	.05	0.03	.24
Exposure to shock	0.32	<.001	−0.08	.01	0.15	<.001
Mental health	0.19	<.001	0.03	.16	1	−
Adherence	−0.12	<.001	1	−	0.03	.16
Physical health	0.21	<.001	0.01	.82	0.31	<.001
WHODAS	1	–	−0.12	<.001	0.19	<.001
CD4 count	−0.00	.72	0.04	.18	−0.05	.06
BMI	0.01	.54	0.04	.14	−0.02	.41

Independent variables with a *p*-value ≤ .25 were included in the linear regression model (Bursac, Gauss, Williams, & Hosmer, 2008). The variables were log-transformed to ensure data linearity. Age, gender, education, marital status, income, exposure to shock, physical health symptoms, the duration of HIV from the time of diagnosis, adherence to and duration of the period on ART, and the mental health score were included in the multivariate regression analysis. The multivariate linear regression showed that mental health, physical health symptoms, income and exposure to shock are stronger predictors of functional limitations. On the other hand, the stronger predictors of mental health scores in the study were found to be age, physical health symptoms, functional limitations/disability and the duration of HIV from the time of diagnosis. Gender, income, a disability score and a mental health score were stronger predictors of adherence to ARVs. Table 4 shows the multivariate linear regression results of the association between the independent and dependent variables.

**Table 4.** Multivariate linear regression analysis of the health outcome predictors of disability among PLHIV on ARV >6 months using linear regression.

	WHODAS			Adherence			Mental health		
	*B*	95% CI	*p*	*B*	95% CI	*p*	*B*	95% CI	*p*
Mental health	0.18	0.16–0.38	<.001	−0.02	−0.04–0.02	<.001	–	–	–
Adherence	−0.13	−0.86–(−0.29)	<.001	–	–	–	−0.02	−0.24–0.15	.64
Physical symptoms	0.11	0.06–0.31	<.001	0.03	−0.02–0.04	.45	0.36	0.32–0.47	<.001
WHODAS	–	–	–	–0.16	−0.06–(−0.02)	<.001	0.18	0.07–0.17	<.001
Age	−0.00	−0.39–0.38	.98	0.07	−0.01–0.19	.08	0.10	0.14–0.65	<.001
Gender	0.06	−0.01–0.14	.08	0.10	0.01–0.04	.01	0.03	−0.03–0.07	.35
Education	0.04	−0.02–0.09	.21	−0.02	−0.02–0.01	.57	−0.03	−0.05–0.02	.39
Marital status	−0.01	−0.04–0.03	.68	−0.02	−0.01–0.01	.60	−0.01	−0.02–0.02	.83
Income	0.13	0.13–0.41	<.001	0.21	0.07–0.14	<.001	−0.07	−0.19–0.00	.05
Exposure to shock	0.28	0.20–0.33	<.001	−0.04	−0.03–(−0.01)	.37	0.06	−0.01–0.08	.10
Number of years living with HIV from time of diagnosis	0.02	−0.14–0.22	.64	−0.08	−0.08–0.01	.15	−0.11	−0.25–(−0.02)	.02
Years on ARVs	0.05	−0.08–0.28	.27	0.00	−0.04–0.05	.94	0.04	−0.06–0.17	.35

Furthermore, the multivariate results in Table 5 show the health predictors of functional limitation in the six domains, namely mobility, cognition, self-care, life activity, getting along and participation after adjusting for age, gender, education, marital status, income, exposure to shock, the duration of HIV from the time of diagnosis and the duration of the period that the patient was on ART.

**Table 5.** Multivariate regression analysis of health outcome predictors of disability among PLHIV on ARV >6 months using linear regression with respect to the WHODAS domains.

	Adherence			Mental health		
	*B*	95% CI	*p*	*B*	95% CI	*p*
WHODAS score	−0.13	−0.87–(−0.30)	<.001	0.18	0.16–0.38	<.001
Mobility	−0.09	−0.45–(−0.06)	.01	0.19	0.11–0.26	<.001
Cognition	−0.10	−0.38–(−0.07)	.01	0.17	0.07–0.19	<.001
Participation	−0.07	−0.35–(−0.01)	.04	0.16	0.07–0.20	<.001
Self-care	−0.13	−0.28–(−0.08)	<.001	0.10	0.01–0.08	.02
Getting along	−0.28	−0.58–(−0.35)	<.001	0.09	0.01–0.09	.02
Life activity	−0.11	−0.34–(−0.08)	<.001	0.19	0.07–0.17	<.001

A one unit increase in the mental health score increased the WHODAS score (functional limitation) by 0.18. In the specific domains, a unit increase in the mental health score increased the WHODAS score in the mobility, cognition and participation domains by 0.19, 0.17 and 0.16, respectively. Also, a one unit decrease in the adherence score reduced the WHODAS score by 0.13. In the specific domains, a unit increase in the adherence score reduced the WHODAS score in the getting along, self-care and life-activity domains by 0.28, 0.13 and 0.11, respectively.

## Discussion

This study assessed the impact and predictors of HIV-related functional limitations/disability and health outcomes in a cohort of PLHIV on ART for more than six months. In this cohort, 51.9% of the participants scored 2 or more on the WHODAS 2.0 scale, using the adjusted measure (Hanass-Hancock & Nixon, 2009; Nyirenda et al., 2012; Scholten et al., 2011). This is higher than in the sister study in KZN (Hanass-Hancock et al., 2015) and suggests a high prevalence of functional limitations that, if not addressed, may pose a risk for disability. In many studies, disability is described differently and often interchangeably between functional and activity limitation, disability and functioning (Myezwa et al., 2009; Nyirenda et al., 2012; Peltzer & Phaswana-Mafuya, 2008; Scholten et al., 2011). Current measures of disability such as the WHODAS 2.0 or the Washington set of questions (Center for Disease Control and Prevention, 2006) make use of this interpretation and measure functional limitations to understand disability. This method identifies people with more moderate conditions that can be addressed with therapeutic interventions as opposed to those with severe conditions that need complex rehabilitative interventions. In this cohort, the levels of functional limitation/disability remain high, and a large part of this population has more moderate limitations that are also less visible. One difference between the current study and previous studies is that the latter focused on older people (Nyirenda et al., 2012; Peltzer & Phaswana-Mafuya, 2008; Scholten et al., 2011) living with HIV, while 99.7% of the cohort from this study were from the economically active age group (20–60). Comparably, Rusch et al. (2004) reported a higher prevalence of disability in their study of an HIV population. There is also a difference in the gender distribution of functional limitations/disability in the cohort for this study and this is consistent with other studies. Females are more prone to report disability than males (Banks et al., 2015; Hanass-Hancock et al., 2013, 2015; Olley, Seedat, Nei, & Stein, 2004; Scholten et al., 2011).

Our study showed that levels of functional limitations/disability are higher in the areas of mobility, cognition and participation and life-activity domains. Similarly, Nyirenda et al. (2012) reported mobility, as well as participation and household activities, as the most affected domains in their study of an older population with HIV. Difficulties in mobility are anticipated as HIV is known to affect the musculoskeletal system negatively. For example, peripheral neuropathy directly affects muscle function, as well as aptotically affecting the muscle cells (Goletz et al., 1997; Miró et al., 2005). Clearly, a large number of our cohort reported mobility limitations.

In our study, adherence to ARVs showed an inverse relationship to overall disability and this is true in all six of the WHODAS 2.0 domains. Similar results were reported in the sister cohort study in KZN (Hanass-Hancock et al., 2015). The HIV-Live Study is the first study to measure the direct association between disability (as a functional limitation) and adherence. The role of adherence to ARVs in the progression of HIV and the occurrence of related co-morbidities has been reported in several studies (Lima et al., 2007; Tantisiriwat et al., 2009; Zolopa et al., 2009). Adherence to ARV therapy reduces or delays the progression of HIV to AIDS and reduces the risk of co-morbidities (Lima et al., 2007; Zolopa et al., 2009). At this point, we are unable to fully understand the direction of this relationship. It is, however, plausible that the lack of adherence to treatment increases the risk of opportunistic infections and co-morbidities and therefore disability. It is also plausible that disability negatively impacts on health-seeking behaviour and therefore adherence to treatment. Much work has been done on adherence to ARVs; however, no known study has been conducted to examine to what extent adherence is related to the onset of disability.

The physical health state of participants was also found to be associated with disability. A poorer state of health is often directly associated with a decreased level of function in the general population (Geyh, Cieza, Kollerits, Grimby, & Stucki, 2007; Woby, Roach, Urmston, & Watson, 2007). In addition, adherence to ARVs has been shown to improve the overall health status of PLHIV (Lima et al., 2007; Zolopa et al., 2009). In this cohort, as well as its sister study (Hanass-Hancock et al., 2015), there is a relationship between adherence and functional limitations/disability. Similarly, in both studies depression correlated positively with increasing disability and all the subcategories of the WHODAS2.0 scores. A discussion on the implications of depression and disability is beyond the scope of this paper, but a matter for further analysis and publication.

Preventing and managing disability is important as it impacts on the productivity of a community, the functioning of its members, and their health-related quality of life (HRQOL) (Chetty, Hanass-Hancock, & Myezwa, 2016). In practice, the management of disability is both multi-sectoral and person-centred (Chetty et al., 2016).

The response to the needs of PLHIV could be divided into micro (person), meso (community) and macro (policy). At the personal level, functional limitations can be managed to mitigate the impact on individuals’ functioning in their daily life through interventions with a rehabilitation team, including physiotherapists, occupational therapists, speech therapists and social workers.

At the meso level, further studies are needed to explore the specific details of mobility, cognition and life participation. Community-based interventions at the meso level are implemented through specific evidence-based interventions to mitigate functional limitations experienced by PLHIV, and hence are important for effectiveness. The results reflect nuanced effects of functional limitations/disability that are similar to disability in the general population but also exhibit important differences peculiar to HIV. Of interest are the relationships between functional limitations, adherence and mental health and participation-related problems.

Finally, at the macro level, the impact of HIV-related disability on policy is important to consider. For example, in the last South African National Strategic Plan for STIs, HIV and TB for 2012–2016, disability was included in two areas, namely those of wellness and health and the provision of services to PWD (South African National AIDS Council, 2012). There was, however, no mention of the need to include rehabilitative services. The new South African National Strategic Plan on HIV/TB/STIs 2017–2022 has recently recognised the need to address the new health-related needs of people living over the long term with chronic HIV or TB and therefore included rehabilitation into the framework (South African National AIDS Committee, 2016).

The results of this study can be used to inform the implementation of the ambitious new plan through providing a better understanding of the link between disability, adherence and mental health (depression), respectively. In a similar vein, the new South African Framework and Strategy for Disability and Rehabilitation Services (2015–2020) clearly states that HIV is a major cause of the prevalence of increased disability in South Africa and calls for the integration of services (Department of Health, 2016). The results of this study could provide a starting point to discuss potentially needed services.

The relationship between HIV and disability also has the potential to impact on the current targeted outcomes in important policy statements such as the 90:90:90 UN statements that states that ‘by 2020, 90% of all PLHIV will know their HIV status, 90% of all people with diagnosed HIV infection will receive sustained antiretroviral therapy (ART), 90% of all people receiving ARTs will have viral suppression’ (UNAIDS, 2014a). Thus the need to integrate services has been recognised by the 2016 WHO’s Consolidated Guidelines on Antiretroviral Treatment (World Health Organisation, 2016). These guidelines acknowledge the need to integrate palliative and rehabilitative services. How this can be implemented within the global response to HIV and AIDS still needs to be outlined more clearly.

## Limitations of this study

A limitation in this study was the inclusion criteria, which included people on ART for six months or longer. That the line of ART was not considered could have affected the results of this study concerning the prevalence and the intensity of disability in the study population. Furthermore, although data on common symptoms were collected, additional data on co-morbidities would have added value to enhance the understanding of the factors associated with disability and HIV.

## Conclusions

The large number of people in South Africa on ART who are experiencing functional limitations and their impacts on other health outcomes in this cohort highlights the need for a continuum of HIV care that prevents and mitigates disability. Wellness for PLHIV will not be complete without the inclusion of rehabilitative services that can prevent and manage known disability. Conversely, the provision of services for people with disabilities (e.g. assistive devices, therapy and environmental accessibility) is closely tied to these efforts of addressing disability among PLHIV as a consequence of HIV. Hence the respective aspects of preventing and accommodating disability need to become a central part of managing the experience of living with HIV over a life-time. In addition, the impact of HIV on PWD is similar to that in other population groups impacted by HIV. However, it is important to consider that they may be exposed to the problems that come with having to deal with disability alone but that this is further compounded by the problem of having a disability and suffering from HIV.

## Notes

1 The ICF is a system for classifying the health components of functioning and disability.

2 Since 2004, over 140,000 patients in the Themba Lethu Clinic have been introduced to antiretroviral medication (ARVs), and currently there are 8000 patients on treatment.
